# Ketamine enhancement of dexmedetomidine attenuation of methamphetamine-induced agitation in rats

**DOI:** 10.3389/jpps.2026.16294

**Published:** 2026-04-15

**Authors:** Madhuri Budamkayala, Jyostna Yalakala, Madison B. Skeen, Surya Karuturi, Chelsey McPhillen, Kristen Bailey, Todd H. Davies, Michael D. Hambuchen

**Affiliations:** 1 Department of Pharmaceutical Sciences, Marshall University School of Pharmacy, Huntington, WV, United States; 2 Marshall Toxicology, Marshall Health, Scott Depot, WV, United States; 3 Department of Family and Community Health, Marshall University Joan C. Edwards School of Medicine, Huntington, WV, United States

**Keywords:** agitation, dexmedetomidine, ketamine, methamphetamine, preclinical, sedation, locomotor, rat

## Abstract

Methamphetamine (METH)-induced agitation, a major concern in acute METH intoxication, is currently treated with benzodiazepines. Due to current polysubstance use patterns in METH consumption, this treatment may fatally exacerbate respiratory depression produced by opioid adulterants or intentionally co-administered opioids. We previously showed that the α2-agonist dexmedetomidine (DEX), which does not potentiate opioid-induced respiratory depression in clinical practice, can be safely and effectively co-administered with naloxone to attenuate METH-induced agitation following naloxone reversal in METH-fentanyl co-intoxicated rats. While the unique arousability of DEX-induced sedation is clinically useful, the current study tested the safety and efficacy of DEX and adjunctive ketamine (KET) in producing deeper, less arousable sedation when needed (i.e., for severe agitation or to facilitate an intricate procedure). Fifteen minutes after 1 mg/kg METH administration in male rats (simulating treatment of naloxone-unmasked agitation with a delay), low-dose (0.032 mg/kg) DEX ± (56 mg/kg) KET, high-dose (0.18 mg/kg) DEX, or saline was administered. Key measurements included METH-induced locomotor activity (a rat model of agitation), the rat coma scale (a quantification of arousability), and α_2_-agonist class side effects. Both high-dose DEX and DEX-KET almost completely attenuated METH-induced locomotor activity for 90 min after administration, but with the combination the sedation was deeper during the most intense METH-induced stimulation, and the α_2_-agonist side effects were less intense and of shorter duration. These data provide proof-of-concept support for the potential use of DEX-KET in producing deeper sedation in METH-induced agitation.

## Introduction

Illicit polysubstance use, which includes the concurrent administration of stimulants (commonly methamphetamine [METH]) and opioids, has increased in recent years [1, 2]. This is due to both adulteration [3, 4] and intentional attempts to enhance or modulate the effects of either class [5, 6]. METH-induced agitation is a major concern in the management of acute METH toxicity because the associated excessive and potentially aggressive patient movements pose a serious hazard to both patients and clinicians [7]. This agitation is currently treated with DEA Schedule IV benzodiazepines (e.g., diazepam) [7], but with the current trend of METH-opioid co-use, the administration of benzodiazepines is concerning, as the boxed warnings for both opioids and benzodiazepines state that “concomitant use of benzodiazepines and opioids may result in profound sedation, respiratory depression, coma, and death” [8, 9]. While naloxone reverses the effects of opioids, an opioid-benzodiazepine interaction may occur with incomplete opioid reversal [10]. This may be due to inadequate naloxone administration or naloxone's more rapid elimination compared with commonly misused opioids such as fentanyl [11] or heroin and its active metabolites [12]. Therefore, alternative sedatives for METH-induced agitation are needed.

Dexmedetomidine (DEX) is a non-DEA-scheduled α_2_-agonist sedative indicated for use in surgical procedures [13] and for the treatment of agitation in schizophrenia and bipolar disorder [14]. Limited clinical case report data show that DEX is effective in treating METH-induced agitation (even after benzodiazepines have failed) [15] and agitation resulting from other stimulants [16, 17]. It may be particularly suited for METH-induced agitation in cases of polysubstance exposure, as it produces minimal respiratory depression even when opioids are also present in humans [18–21]. Opioids are known to mask stimulant-induced agitation, which becomes unmasked once the opioid effects subside (i.e., due to pharmacokinetic clearance or rapid reversal with naloxone) [10]. Using METH-induced locomotor activity as a rat model of METH-induced agitation [22–24], we provided initial proof-of-concept that DEX-naloxone co-administration safely reverses fentanyl-induced sedation while potently, significantly, and substantially attenuating the unmasked METH-induced agitation in male rats [25].

DEX also produces sedation in which the patient is arousable (i.e., more like natural sleep) and can respond to simple commands [18, 19]. This unique sedation could effectively treat METH-induced agitation and use hospital resources more efficiently, as deep sedation is significantly correlated with more prolonged hospital stays [26]. Deeper sedation may be needed, however, in severely agitated patients or to facilitate the performance of more intricate procedures (e.g., implanting an intravenous line) upon presentation. Due to both the arousability of sedation [19] and the presence of α_2_-agonist side effects, aggressive DEX dosing to achieve this deeper sedation may be inadvisable. Due to sympatholytic effects mediated through the α_2a_-receptor, DEX initially causes hypotension and bradycardia [27]. With increasing DEX concentrations, heart rate is further reduced while blood pressure increases through α_2b_-receptor-mediated vasoconstriction. DEX binds α_2_-receptors on pancreatic cells to inhibit insulin secretion and increase glucagon secretion, potentially resulting in hyperglycaemia [28]. DEX also causes thermal dysregulation and potentially hypothermia in humans by lowering the temperature threshold required for compensatory shivering and vasoconstriction, which generate and conserve heat, respectively [29].

Adjunctive ketamine (KET) with DEX may be useful for safely providing this deeper sedation without increasing the DEX dose and therefore the incidence of dose-dependent adverse effects. KET is a dissociative anaesthetic NMDA antagonist with additional sedative and analgesic effects through partial µ-opioid receptor agonism [30]. Combination DEX-KET has previously been used to enhance sedation in animal research [31, 32] and veterinary practice [33, 34] and is increasingly being used in clinical medicine [35–38]. KET may provide additional advantages in cases of polysubstance exposure, as it, similar to DEX, causes minimal respiratory effects [39]. KET also increases heart rate, blood pressure, and cardiac output through the activation of the sympathetic nervous system [40], which could attenuate the bradycardic and hypotensive effects of DEX [27].

For this study, we elected to focus on the interaction between DEX and KET in safely and effectively attenuating METH-induced agitation and therefore did not include fentanyl-METH co-administration and naloxone reversal in combination with DEX ± KET treatment. In addition, this study design provided a more rigorous test of the combination in the context of ongoing METH-induced locomotion. This is in contrast to our initial DEX-NLX study, in which locomotor activity was inhibited as it was more gradually unmasked from fentanyl-induced sedation by naloxone [25]. The current study, therefore, simulates a clinical scenario in which DEX ± KET is administered after naloxone unmasks agitation in a fentanyl-METH co-intoxicated patient. In humans, this would most likely occur when or before peak naloxone concentrations are reached, typically within 15–30 min of intranasal administration [41]. We hypothesised that the combination of low-dose DEX with adjunctive KET could produce a more intense, less arousable early sedation in METH-intoxicated rats than high-dose DEX, with a superior adverse effects profile.

Preliminary studies followed by a dose-verification study (Supplementary Material 1) were performed to determine and verify, respectively, the doses administered in this study. This included the minimally effective dose of DEX (0.032 mg/kg, “low-dose”) needed to significantly reduce 1 mg/kg METH‐induced locomotor activity, the high‐dose of DEX (0.180 mg/kg), and the adjunctive dose of KET (56 mg/kg) with low‐dose DEX required to almost completely attenuate this activity during the first 90 min after treatment. This time interval was of interest as it includes the most intense METH-induced activity. These preliminary and dose-verification studies showed that treatment with KET alone resulted in enhanced early METH-induced stereotypy followed by a more intense period of locomotor activation than produced by METH alone. Therefore, we chose to exclude the KET monotherapy group from the study. In the preclinical trial, we first tested the ability of low-dose DEX ± KET and high-dose DEX to attenuate 1 mg/kg METH-induced locomotor activity over both the key 90-min post-treatment period and for 4 h after treatment. Approximately 1 week later, we repeated the METH and treatment doses to test the arousability of sedation and the α_2_-agonist side effect profile over the same 4-h period, using methods previously developed by our research group [42]. Renal clearance of unchanged METH is a contributor to METH elimination in both rats [43] and humans [44], and bolus DEX administration is known to enhance urine formation [45, 46], likely via reduced vasopressin release [47, 48]. Urine was collected between measurements during the sedation arousability/side effects trial to evaluate potential DEX-induced changes in renal METH clearance (i.e., through urinary dilution producing a reduced METH gradient for reabsorption) [49, 50].

## Materials and methods

### Drugs

A 1 mg/kg METH ([S]-methamphetamine HCl; Sigma-Aldrich, St. Louis, MO, USA) solution was prepared in saline (SAL) for subcutaneous (SC) administration at 1 mL/kg and stored under refrigeration to maintain stability [51]. DEX hydrochloride (veterinary grade, 0.5 mg/mL; Dechra, Cheshire, CT, USA) was diluted in SAL to 0.032, or 0.18 mg/mL for SC administration at 1 mL/kg (i.e., 0.032, or 0.18 mg/kg, respectively). KET HCl (veterinary grade, 100 mg/mL; Covetrus, Portland, ME, USA) was diluted with SAL to 56 mg/mL for SC administration at 1 mL/kg (i.e., 56 mg/kg). The DEX and KET dilutions were prepared just prior to each experiment, as the stability of the diluted agents is unknown.

### Animals

Male Sprague Dawley rats (Hilltop Laboratory Animals, Scottsdale, PA, USA; RRID:RGD_25824850) weighing 261 ± 9 g and approximately 7 weeks old on study day 0 (n = 32, 8/treatment group) were used. The rats were housed in pairs in a room maintained at 21–22 °C and 40–55% humidity with food and water provided *ad libitum*. The animal use protocol was approved by the Marshall University Institutional Animal Care and Use Committee (Protocol #855) and was performed in compliance with the National Institutes of Health (NIH) Guide for the Care and Use of Laboratory Animals and the ARRIVE guidelines.

### Overall design

A summary of the experiments performed and the location of the resulting data in the manuscript is provided in Table 1. The laboratory in which the experiments were performed was maintained at approximately 24 °C. On days −5 and −4, the rats were conditioned to the researchers and gently restrained (i.e., for injections and measurements of blood glucose and temperature) by being handled in towels intended for use in each cohort of the study. On day −1, the rats were SC injected with 1 mL/kg SAL prior to the measurement of 14 min of locomotor activity. The rats were then SC injected with SAL at two sites prior to the measurement of 1 additional hour of locomotor activity, starting at 0 min. The locomotor study was performed similarly on day 0, with the SC injection of 1 mg/kg METH prior to SC SAL injections at two sites, followed by the measurement of 240 min of locomotor activity. The 1 mg/kg METH dose was chosen as it produces a robust locomotor effect with minimal stereotypy in rats, which is not significantly different from the effect produced by 1 mg/kg intravenous METH [52]. The initial 90-min post-treatment administration period was targeted to induce deep sedation, as it comprised the most intense locomotor activity produced by 1 mg/kg METH in rats in the preliminary studies. METH-induced locomotor activity measured during this 90-min interval was used to group the rats according to their average METH-induced activity levels.

**TABLE 1:** Experimental overview, including schedule, groups, and experiments performed.

Day	Procedure Performed
−5 and −4	Rats conditioned to handling while wrapped in the towels used for gentle restraint during injections and blood glucose/temperature measurements
−1	SAL locomotor activity: conditioning • Rats injected with SAL once at −15 min and twice at 0 min • Locomotor activity measured between injections and until 60 min
0	METH locomotor activity: division into groups • Rats injected with METH once at −15 min and SAL twice at 0 min • Locomotor activity measured between injections and until 240 min • Total distance travelled data from 90 min post–saline used to divide the rats into treatment groups with almost identical average activity • Outcomes: Distance travelled over time (Figure 1A) and total distance travelled (Figure 2A pre–SAL; Figure 2B 90 min post−SAL; Figure 2C 240 min post−SAL)
1	METH locomotor activity: treatment efficacy • Rats injected with METH once at −15 min and (1) SAL or DEX followed by (2) either KET or SAL (in non−KET−treated groups) at 0 min to generate the following post−METH control or treatment groups (n = 8/group): • SAL (control) • *Low−dose DEX (0.032 mg/kg)* • *Low−dose DEX (0.032 mg/kg) + KET (56 mg/kg)* • *High−dose DEX (0.18 mg/kg)* • Locomotor activity measured between injections and until 240 min • Outcomes: Distance travelled over time (Figure 1B) and total distance travelled (Figure 2D pre−treatment; Figure 2E 90 min post−treatment; Figure 2F 240 min post−treatment)
2	Visual evaluation of the condition of the rats and post−treatment weight • Outcome: weight changes (details are in the post−treatment weight section in the results)
7 or 8	Arousable sedation/side effects study • Repeat day 1 drug exposures and place rats into diuresis cages for urine collection until 240 min • Outcomes: Urine volume recorded prior to storage at −80 °C (Figures 5A,D); Urine pH (Figures 5B,E) and % unchanged METH (Figures 5C–E) determined by collaborating clinical toxicologists • Rats removed at 20, 60, 120, 180, and 240 min after treatment or control administration for a battery of tests • Outcomes (sedation arousability): rat coma scale, need for restraint during blood glucose and temperature measurements (Figure 3) • Outcomes (side effects): SpO2 (Figure 4A), heart rate (Figure 4B), blood glucose (Figure 4C), temperature (Figure 4D)

On day 1, the locomotor study was repeated with four groups administered either low-dose (0.032 mg/kg) DEX, low-dose DEX +56 mg/kg KET, high-dose (0.18 mg/kg) DEX, or SAL control 15 min after METH administration (n = 8/group). DEX doses were determined based on preliminary studies (0.01 – 0.18 mg/kg), which showed that 0.032 mg/kg DEX significantly reduced 1 mg/kg METH-induced locomotor activity, while 0.18 mg/kg DEX almost completely eliminated activity during the 240-min trial. This was followed by a preliminary study of adjunctive KET (10 – 100 mg/kg) in combination with 0.032 mg/kg DEX. This confirmed that the addition of 56 mg/kg of KET produced the most prolonged reduction in activity with the least post-sedation KET emergence response. Since the administration of 56 mg/kg of KET 15 min after METH and in the absence of DEX resulted in intense stereotypy followed by an enhanced peak locomotor effect, a KET-alone treatment group was excluded from the study. The methods and results of the small locomotor study (n = 4/group) performed to qualitatively verify the locomotor attenuating effects of the chosen DEX ± KET doses are reported in Supplementary Material 1. Treatment doses were prepared by a separate researcher to blind the researcher performing the rat coma scale scoring to the treatment administered. DEX and KET were administered separately at distinct sites due to poor compatibility in solution [53]. SAL was injected in place of KET in the non-KET-treated groups to maintain blinding for coma scale scoring on day 7 or 8 and to ensure a consistent number of injections between groups. After treatment, 240 min of activity were measured.

On experimental day 2, the rats’ condition was evaluated, and their weights were recorded. The percentage weight change compared to pre-drug exposure weights on day 0 was calculated by subtracting each rat’s post-treatment weight on day 2 from its pre-drug exposure weight, dividing by the pre-drug exposure weight, and multiplying by 100.

Drug exposure was repeated in the same groups of rats for the arousable sedation/adverse effect study on day 7 or 8. This study was performed on two separate days due to its extended duration and the inclusion of multiple timed measurements for each rat. To account for any potential effect of the time between days 7 and 8, one rat from each group was tested on each day. After METH and treatment administration, each rat was placed in a diuresis cage for urine collection between measurements during the 4-h trial. Each rat was then removed from its diuresis cage for the measurement of arousable sedation (rat coma scale), heart rate, saturation of peripheral oxygen (SpO2), blood glucose, and temperature 20-, 60-, 120-, 180-, and 240-min after treatment administration. Baseline values for each measurement were collected shortly before METH administration. Prior to baseline SpO2 and heart rate measurements, the rats were conditioned to a standard bedding-free rat cage (one cage per animal with bedding removed to maintain clear airways in sedated rats). This cage was used to position the rats for the measurement of SpO2 and heart rate and for the transport of rats between tests.

### Locomotor activity

Horizontal locomotor activity was measured as the distance travelled in open-field polyethylene chambers (74 cm tall with a 58 × 58 cm base) using overhead cameras interfaced with the Noldus EthoVision 14 automated behavioural analysis system (Noldus Information Technology Inc, Sterling, VA, USA; RRID:SCR_000441). Data were output as distance travelled (M) in 1-min intervals for each rat to assess qualitative patterns of locomotor activity over time in 5-min intervals, total post-METH/pre-treatment distance travelled, total 90-min post-treatment distance travelled, and total 240-min post-treatment distance travelled.

### Arousable sedation

Arousable sedation was primarily measured with an adaptation of the previously validated rat coma scale, which was based on the Glasgow coma scale and other similar neurological assessment tools used in humans [54]. After being gently removed from the diuresis cage, each rat was placed on a flat bench surface for scoring of the following: (1) spontaneous whisker movement, (2) motor function, (3) brain stem reflexes, (4) righting reflex, and (5) auditory startle response, as described previously [42] (a summary is provided in Table 2). A score out of 10 was reported for each rat. In addition, the observation of a rat not requiring restraint in a towel for blood glucose and temperature measurements was recorded as a secondary qualitative measurement of deeper sedation.

**TABLE 2:** Rat coma scale scoring. It should be noted that the corneal reflex is present if the rat blinks in response to its eye being gently touched with a sterile cotton swab soaked in sterile saline, while the pinna reflex is present if the rat shakes its head in response to a flexible monofilament gently placed into its ear.

Total score: 0 – 10	Score 0	Score 1	Score 2	Score 3	Score 4
(1) Whisker movement	No movement	Spontaneous movement	​	​	​
(2) Motor function	No response to paw pinch	Fasciculations in response to paw pinch	Walks or deliberately withdraws paw due to paw pinch	Walks or deliberately withdraws paw due to paw touch	Walks voluntarily when placed on a flat surface
(3) Brain stem reflexes	Neither reflex present	Has EITHER corneal or pinna reflex	Has BOTH corneal and pinna reflex	​	​
(4) Righting reflex	No righting	Partial righting (rolls to side)	Full righting	​	​
(5) Auditory reflex	No response to startle	Auditory startle (to a clap above head)	​	​	​

### SpO2 and heart rate

Rats were placed in a bedding-free cage, to which they had previously been acclimatised, for the measurement of SpO2 (%) and heart rate (beats per minute [BPM]) with a properly sized collar sensor interfaced with a MouseOx Plus pulse oximeter (STARR Life Sciences Corp., Oakmont, PA, USA). This sensor type facilitated measurements in both conscious and sedated rats. Upon proper initialisation of the pulse oximetry signal, 10 s of stable SpO2 and heart rate readings were recorded and reported as an average value for each rat.

### Blood glucose

Rats were moved from the bedding-free cage to a towel that had been previously used during the conditioning sessions on days −5 and −4. If the rat moved upon placement on the towel, during the cleaning of the tail, or during the collection of blood or temperature measurements, the rat was gently restrained (it was noted when no restraint was required; see “Arousable Sedation” above). The tail was cleaned with 70% isopropyl alcohol and wiped dry with gauze before a light puncture was made with a 27 G needle to collect a small drop of blood (i.e., <10 µL). A test strip interfaced with a consumer-grade Ascensia CONTOUR NEXT portable blood glucometer (Parsippany, NJ, USA) was applied directly onto the drop to measure blood glucose, followed by replacement of the strip and collection of a duplicate measurement. Due to the small volume, the majority of collections after baseline could be completed by cleaning the tail and gently massaging a small drop of blood from the previous puncture site.

### Temperature

Temperature measurements were performed with a water-soluble, non-toxic lubricant-coated rectal probe sized for rats, interfaced with a research-grade thermometer (Braintree Scientific, Braintree, MA, USA) while the rat was still restrained, if needed for blood glucose measurements. When the reading stabilised and remained constant for 5 s, the temperature was recorded. After each measurement, the probe was cleaned with 70% isopropyl alcohol.

### Urine collection and analysis

After treatment administration at 0 min, each rat was placed in a Tecniplast diuresis cage (West Chester, PA, USA) for 240 min of urine collection between measurements. Any urine loss observed during measurements or transportation between measurements was noted, and urine voided into the bedding-free pulse oximetry/transport cage was collected with a transfer pipette. After measuring the total volume of collected urine, an approximately 1 mL aliquot was stored at −80 °C for analysis.

Urine pH was measured with a CLC1600 chemistry analyser using a reagent kit, calibrators, and quality control solutions provided by the vendor (Carolina Liquid Chemistries, Greensboro, NC, USA). Urine pH was measured spectrophotometrically by measuring the changes in absorbance in a colourimetric reaction using acid-base indicator dyes, which produce colour depending on the pH of the specimen. A two-point calibration curve using calibrators at pH 3.0 and 11.0 was utilised, and controls at pH 3.6 and 10.0 were processed with every batch.

Prior to extraction for urine METH quantitation, a solid-phase extraction mix containing internal standards and B-One® room temperature β-glucuronidase (Kura Biotech, Atlanta, GA, USA) was prepared. For each row of the 96-well plate being extracted, 600 µL of the internal standard stock solution containing 500 ng/mL of methamphetamine-d11 was combined with 3 mL of β-glucuronidase. Solid-phase extraction was performed using NBE HPSCX 96-well extraction plates (Tecan, Baldwin Park, CA). A total of 250 µL of urine was combined with 300 µL of the SPE mix and incubated at room temperature for 15 min. The plates were then conditioned with 500 µL of methanol and 500 µL of water containing 0.1% formic acid before loading 500 µL of the specimen/solid-phase extraction mix into the wells. The plates were placed under vacuum, and the specimens were allowed to flow through at a rate of approximately 1–2 mL/min. The wells were then washed with 300 µL of 0.1 M hydrochloric acid, after which the drugs were eluted into 2 mL 96-well plates with 300 µL of 5% ammonium hydroxide in methanol at a rate of 1–2 mL/min. The eluate was dried under nitrogen at 60 °C until dry. The specimens were reconstituted with 200 µL of a 90:10 water: acetonitrile mixture containing 0.1% formic acid, after which they were placed on an orbital shaker at 160 rpm for 6 min.

Quantitation of METH was performed on an Acquity UPLC coupled to a Xevo Tandem Quadrupole Mass Spectrometer (Waters Corporation, Milford, MA, USA; RRID:SCR_018510). A 10 µL specimen was injected, and separation was achieved using a 100 × 2.0 mm 1.6 µm Polar C18 Luna Omega column (Phenomenex Inc., Torrance, CA, USA), which was maintained at 50 °C. The aqueous mobile phase (solvent A) contained 0.1% aqueous formic acid, and the organic mobile phase (solvent B) contained acetonitrile with 0.1% formic acid. The total run time was 6.5 min. The liquid chromatography gradient is shown in Table 3.

**TABLE 3:** Liquid chromatography gradient of mobile phase solvent A (0.1% aqueous formic acid) and solvent B (acetonitrile containing 0.1% formic acid).

Time (min)	Flow (mL/min)	%A	%B
Initial	0.5	98.0	2.0
4.9	0.6	46.9	53.1
5.0	0.6	0.0	100.0
6.0	0.6	98.0	2.0

Quantitation of METH was achieved in positive ionisation mode with a capillary voltage of 1.2 kV. The desolvation temperature was 600 °C, with a nitrogen desolvation gas flow of 1000 L/h. The argon collision gas flow was set to 50 L/h. Data were collected in multiple reaction monitoring (MRM) mode, with the ion transitions monitored for METH and its internal standard, methamphetamine-d11, reported in Table 4.

**TABLE 4:** Ion transitions for the analyte (both quantifier and qualifier) and the internal standard.

Analyte	Precursor (m/z)	Product (m/z)	Cone (V)	Collision (V)
METH (quantifier)	150.1	91.1	37	14
METH (qualifier)	150.1	119.1	37	6
Methamphetamine–d11 (internal standard)	161.1	127.2	25	16

The lower limits of detection and quantitation for METH were 10 ng/mL, and METH was quantified using a linear calibration curve, prepared in negative urine, which ranged from 10 to 2000 ng/mL. Quality control samples at concentrations of 18, 75, 160, and 675 ng/mL were also included in the analysis.

Renal METH elimination was reported as the percentage of the unchanged METH dose present in the urine. This was calculated by multiplying the urine METH concentration by the volume of urine collected, dividing it by the total METH dose administered (i.e., the mg/kg dose multiplied by the rat’s weight in kg), and then multiplying the result by 100.

### Statistical analysis

Due to unequal standard deviations (SDs) between groups, significant differences between groups in total distance travelled in the 90- and 240-min periods post-treatment on day 1, and in urine volume, were determined with a Welch’s ANOVA test followed by a Dunnett T3 *post hoc* analysis. Significant differences between groups in pre-treatment distance travelled, total distance travelled in the 90- and 240-min periods after day 0 treatment, weight change on day 2, urine pH, and percentage of unchanged METH clearance were determined with a one-way ANOVA with a Holm-Šídák’s multiple comparisons test *post hoc* analysis. Significant differences in rat coma scale scores, SpO2, heart rate, blood glucose, and temperature over time between groups were determined with a repeated measures two-way ANOVA with a more conservative Geisser-Greenhouse Correction (to avoid type I error), followed by a Holm-Šídák’s multiple comparisons test *post hoc* analysis. Due to the non-Gaussian distribution of urine pH and volume (confirmed by Anderson-Darling and Shapiro-Wilk tests), a Spearman’s rank correlation coefficient (ρ, two-tailed) was used to assess the association with the percentage of unchanged METH excreted in urine.

## Results

### METH-induced locomotor activity (days 0 and 1)

**FIGURE 1:**
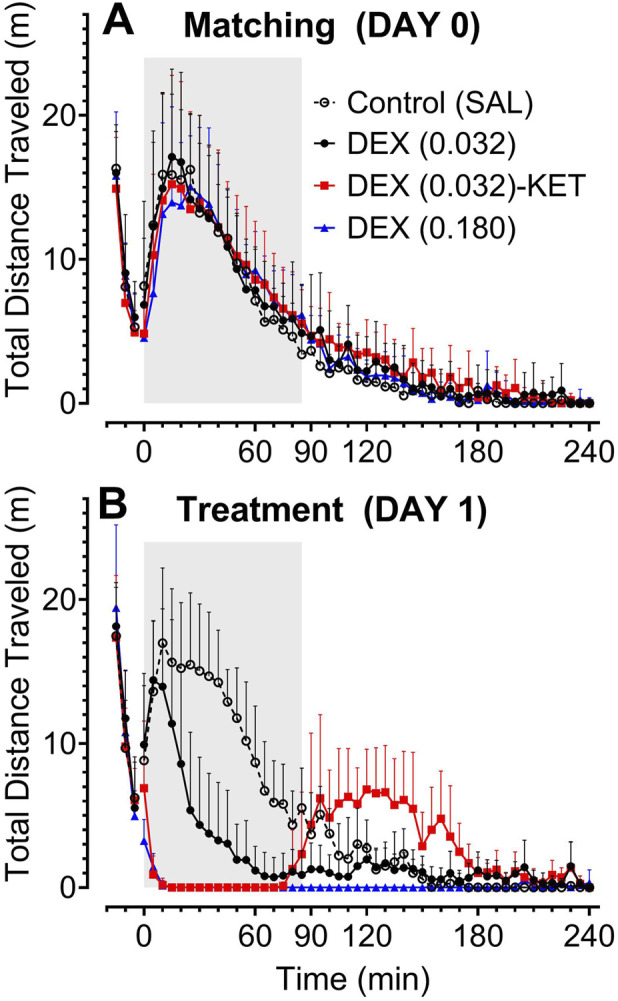
Distance travelled over time (n = 8/group). Average activity over time +SD is plotted in 5–min intervals from METH injection at −15 min to either SAL control **(A)** or SAL or treatment at time 0 **(B)** and for an additional 240 min. The targeted 90–min post–treatment period is highlighted in grey, but only extends to 85 min on the x–axis, as this time point includes data from 85 min up to just 90 min. It should be noted that all rats were treated identically with METH and SAL on day 0 **(A)**, but were divided into treatment groups to visually demonstrate the balance in pre–treatment METH–induced locomotor activity between groups.

**FIGURE 2:**
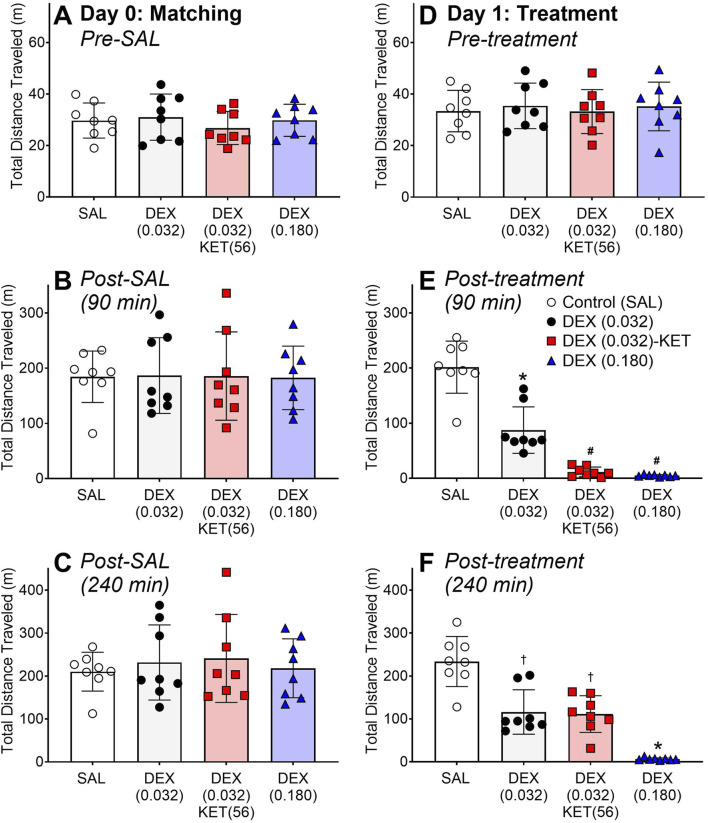
Total distance travelled (n = 8/group). Individual and mean (bar) ± SD METH–induced locomotor activity is plotted prior to **(A,D)**, 90 min after **(B,E)**, and 240 min after **(C,F)** the administration of SAL or treatment at time 0. It should be noted that all rats were treated identically with METH and SAL on day 0 **(A–C)**, but were divided into treatment groups to visually demonstrate the balance in pre–treatment METH–induced locomotor activity between groups. Significant *post hoc* comparisons (p < 0.05) are marked as follows: *significantly different from all other groups, ^#^significantly different from SAL and DEX (0.032), and ^†^significantly different from SAL and DEX (0.180).

The data resulting from the dose verification locomotor activity study are reported in Supplementary Material 1. The overlapping pre-treatment day 0 METH-induced locomotor activity over time profiles (Figure 1A) and the lack of significant differences in total distance travelled data (Figures 2A–C) demonstrate that the rats were effectively divided into treatment groups. As expected, on day 1, pre-treatment METH-induced locomotor activity was similar between groups (Figure 1B [-15–1 min] and Figure 2D). The treatments substantially altered the METH-induced locomotor activity over time profile (Figure 1B [0–240 min]) resulting in significant differences between groups in total distance travelled in both the 90- (Figure 2E; F [3, 12] = 51.95, p < 0.0001) and 240-min (Figure 2F; F [3, 11.74] = 61.29, p < 0.0001) post-treatment periods. Low-dose DEX (0.032) alone significantly reduced METH-induced locomotor activity, while high-dose DEX (0.180) both significantly and almost completely reduced this activity over the entire 240-min trial (Figures 1B, 2E,F). Low-dose DEX (0.032) plus adjunctive KET effectively and almost completely attenuated METH-induced locomotor activity in the targeted 90-min post-treatment period; however, the emergence of post-sedation locomotor activity resulted in a smaller but significant overall effect in the full 240-min trial. Plots for individual rat activity are reported in Supplementary Material 2.

### Post-treatment weight (day 2)

There were no significant weight changes on day 2 (i.e., 24 h after treatment administration on day 1) in the DEX (0.032) (0.08 ± 1.60%) and DEX (0.032)-KET (−1.88 ± 1.69%) groups compared to the SAL control group (−0.36 ± 1.52%); however, weight was significantly reduced (F [3, 28] = 8.274, p = 0.0004) in the high-dose DEX (0.180) (−4.70 ± 3.63%) group.

### Sedation arousability (day 7 or 8)

**FIGURE 3:**
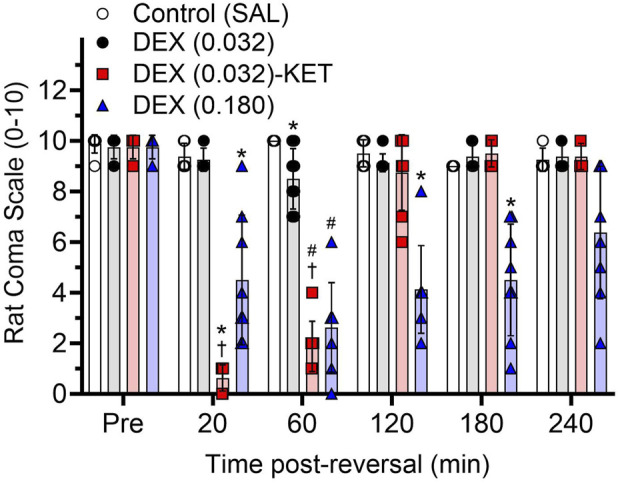
Arousable sedation over time (n = 8/group). Individual and mean (bar) ± SD rat coma scale values before METH administration (Pre) and 20 – 240 min after the administration of SAL or treatment on day 7 or 8. Significant *post hoc* comparisons (p < 0.05) are marked as follows: *significantly different from all other groups and ^#^significantly different from SAL and DEX (0.032). ^†^denotes that a lack of restraint was required for blood collection and temperature measurement as a secondary measurement of arousable sedation.

While there were no pre-treatment differences between groups on day 7 or 8, there were significant time-dependent differences in arousable sedation, as measured by the rat coma scale, post-treatment (Figure 3, F [10.87, 101.4] = 38.47, p < 0.0001). Despite significantly reducing METH-induced locomotor activity (Figures 1, 2E,F), low-dose DEX (0.032)-induced sedation was almost completely arousable, with only a small but significant reduction compared to the SAL group at the 60-min time point. Even high-dose DEX (0.180), which almost completely attenuated the METH-induced locomotor activity (Figures 1B, 2E,F), produced partially arousable sedation in this assay. The low-dose DEX (0.032)-KET combination rapidly produced significantly deeper and less arousable sedation than all other groups 20 min post-treatment. At 60 min after low-dose DEX-KET administration, the rats remained substantially and significantly more deeply sedated than those treated with low-dose DEX alone or the control group (similar to the deepest sedation produced by high-dose DEX, which occurred at this time point) before mean values returned to baseline at 120 min. While all rats treated with low-dose or even high-dose DEX monotherapy required restraint for blood glucose and temperature measurements at all time points, these measurements could be performed without restraint in all 8 low-dose DEX (0.032)-KET-treated rats at both the 20- and 60-min time points after administration.

### Side effects (day 7 or 8)

**FIGURE 4:**
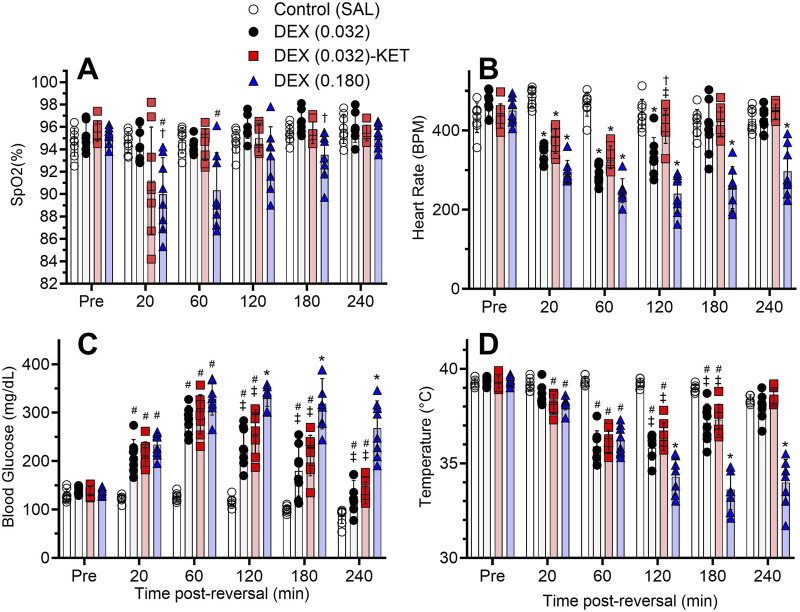
α2–agonist adverse effects over time (n = 8/group). Mean (bar) ± SD SpO2 **(A)**, heart rate **(B)**, blood glucose **(C)**, and temperature **(D)** before METH administration (Pre) and 20 – 240 min after the administration of SAL or treatment on day 7 or 8. Significant *post hoc* comparisons (p < 0.05) are marked as follows: *significantly different from all other groups, ^#^significantly different from SAL, ^†^significantly different from DEX (0.032), and ^‡^significantly different from DEX (0.180).

High-dose DEX (0.180) produced significant reductions in SpO2 compared to the SAL control and/or low-dose DEX (0.032) groups at 20, 60, and 180 min post-treatment (Figure 4A). However, these reductions were not substantial, with full recovery to baseline occurring at the end of the 240-min trial (F [7.693, 71.80] = 3.461, p = 0.0023). All treatments resulted in significant reductions in heart rate (Figure 4B, F [10.52, 98.16] = 17.96, p < 0.0001). High-dose DEX (0.180) produced the most intense reductions, which persisted throughout the 240-min trial. While the less intense but still significant low-dose DEX (0.032)-induced reduction in heart rate recovered to baseline by 180 min, the addition of KET resulted in both a smaller reduction (as evidenced by the significantly higher heart rate compared to low-dose DEX alone between 20 and 120 min) and a more rapid return to baseline by 120 min. There was a substantial and significant increase in blood glucose in all three treatment groups (Figure 4C, F [7.822, 73.01] = 22.63, p < 0.0001). These higher levels, while still significantly elevated compared to the SAL control, largely recovered in the low-dose DEX (0.032) ± KET groups but remained substantially higher in the high-dose DEX (0.180) group at the conclusion of the 240-min trial. There was a substantial and significant reduction in temperature in all treatment groups (Figure 4D, F [8.027, 74.92] = 50.51, p < 0.0001). A minor but significant reduction in temperature was detected 20 min post-treatment in the low-dose DEX (0.032)-KET and high-dose DEX (0.180) groups, with substantial reductions compared to the SAL control group present in all three treatment groups by 60 min. Temperature fully recovered in the DEX (0.032) ± KET groups by the conclusion of the trial, 240 min post-treatment. High-dose DEX (0.180)-induced temperature reductions, however, did not return to baseline and remained significantly lower than all other groups between 120 and 240 min.

### Renal pharmacokinetics (Day 7 or 8)

**FIGURE 5:**
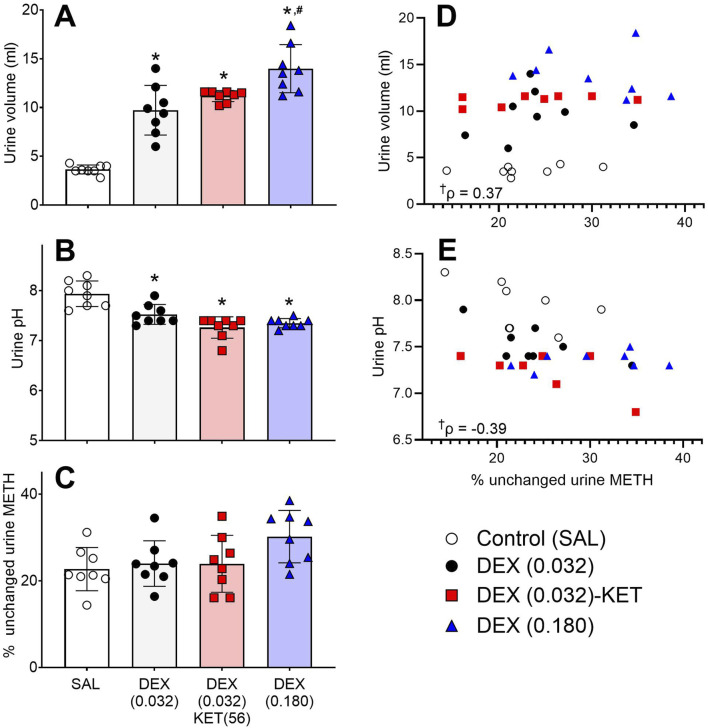
Urine data collected over 240 min (n = 8/group). Mean (bar) ± SD urine volume **(A)**, urine pH **(B)**, percentage of unchanged METH cleared in the urine **(C)**, and individual rat scatter plots for percentage of unchanged urine METH vs. volume **(D)** and vs. pH **(E)**. Significant *post hoc* comparisons (p < 0.05) are marked as follows: *significantly different from SAL and ^#^significantly different from DEX (0.032). While there was no significant change in the percentage of unchanged METH cleared, there was a trend towards a significant effect (p = 0.06). A significant correlation is marked as †.

All three treatments resulted in substantial and significant increases in urine volume (Figure 5A, F [3, 15.15] = 46.46, p < 0.0001) and significant reductions in pH (Figure 5B, F [3, 28] = 18.09, p < 0.001) compared to the SAL control, with high-dose DEX (0.180) significantly increasing urine production compared to low-dose DEX (0.032). While DEX did not significantly enhance the elimination of unchanged renal METH in any group, there was a trend towards increased elimination resulting from the high-dose DEX (0.180) treatment (Figure 5C, F [3, 28] = 2.782, p = 0.0594). There was a moderate, significant positive correlation between the percentage of unchanged METH excreted in urine and urine volume (Figure 5D, ρ = 0.37, p = 0.0351), along with a moderate, significant negative correlation between the percentage of unchanged METH excreted in urine and urine pH (Figure 5E, ρ = −0.39, p = 0.0287).

## Discussion

Low-dose DEX with adjunctive KET produced deeper, less arousable sedation than low-dose and even high-dose DEX at early time points. Despite this intense sedative effect, the side effect profile of the combination was far superior to that of high-dose DEX alone, and it offered some safety advantages over low-dose DEX monotherapy. Future studies will employ this intervention in a METH-fentanyl polysubstance administration scenario with naloxone reversal, as previously performed with DEX-naloxone [25]. The current study, however, serves to emphasise the robustness of the DEX-KET sedative effect by testing it during an intense ongoing METH-induced locomotor response rather than during an emerging response after naloxone reversal of fentanyl-induced sedation. Indeed, the DEX-KET combination is increasingly used for sedation in humans [35–38], but this is the first study to demonstrate the potential utility of DEX-KET pharmacotherapy in treating stimulant-induced agitation.

Low-dose DEX alone was found to significantly reduce METH-induced locomotor activity (Figures 2E,F) by partially attenuating and shortening the duration of the METH-induced effect (Figure 1B). In contrast, high-dose DEX rapidly and almost completely attenuated METH-induced locomotor activity throughout the 4-h trial (Figures 1B, 2E,F). Adding adjunctive KET to low-dose DEX, however, resulted in rapid and almost complete attenuation of METH-induced locomotor activity during the targeted 90-min post-treatment period (Figure 2E). The combination produced an overall reduction in activity similar to that observed with low-dose DEX alone over the full 4-h trial (Figure 2F) due to a post-sedation elevation in locomotor activity (Figure 1B). Emergence reactions, including euphoria, vivid dreams, hallucinations, delirium, dissociation, and agitation [55–57] are common upon recovery from KET-induced sedation [57]. Using locomotor activity as a surrogate measure of agitation in rats [22–24], the enhanced post-sedation locomotor activity in the low-dose DEX-KET group (Figure 1B) could reasonably be interpreted as emergence agitation. Awakening from KET-induced sedation in a minimally stimulating environment reduces the severity of these emergence reactions in humans [57]. Based on our observations on day 7 or 8, rat movement upon awakening in the low-dose DEX-KET group appeared to be less severe in the smaller, enclosed diuresis cages compared to the larger open field on day 1. Nevertheless, this effect could likely be managed by additional administration of low-dose DEX prior to sedated patients’ awakening [58].

The unique sedation provided by DEX is arousable by external stimuli and can facilitate patient response to simple commands during sedation [18, 19]. Despite low-dose DEX significantly reducing METH-induced locomotor activity (Figures 2E,F), the unique sedation produced by DEX resulted in rat coma scale measurements similar to the SAL control group, with only a minor significant reduction at the 60-min time point (Figure 3). Although high-dose DEX produced a prolonged and almost complete attenuation of METH-induced locomotor activity on day 1 (Figures 1B, 2E,F), the sedation was partially arousable throughout the trial (Figure 3). Low-dose DEX-KET, however, significantly reduced arousable sedation compared to even high-dose DEX at the 20-min post-injection time point. It also reduced arousability compared to low-dose DEX and SAL in the 20- and 60-min post-administration measures. Unlike low- or high-dose DEX monotherapy, low-dose DEX-KET facilitated the collection of blood and temperature measurements at these time points without restraint. The intense, early, low-dose DEX-KET-induced sedative effect is notable, given that METH-induced locomotor activity was most intense approximately 20 min after SAL administration in the control group (Figure 1B). In a potential future clinical application of this intervention, clinicians could initially take advantage of the unique arousable sedation of DEX [18, 19] and then subsequently administer KET to deepen this sedation if necessary.

The same side effects occur to some extent with DEX-KET but they are just considerably “less severe” than with high dose DEX monotherapy. Aside from the emergence agitation [57] produced by the combination (Figure 1B), it also offered some safety advantage compared to low-dose DEX. While day 2 post-treatment weight changes were similar in low-dose DEX ± KET-treated rats compared to those treated with SAL, high-dose DEX resulted in significant overnight weight loss of ∼5%. The cause of this weight loss is unclear, as α_2_-agonists can sometimes increase [59–61] or maintain weight [25, 62] while at other times they result in weight loss [42, 63] with chronic administration. Therefore, we interpret the significant reduction in weight after high-dose DEX, but not after low-dose DEX-KET, as further evidence that the combination is more tolerable compared to high-dose DEX monotherapy.

Aggressive administration in the high-dose DEX group resulted in some minor but significant reductions in SpO2 (Figure 4A). Low-dose DEX ± KET, however, did not produce any significant reductions in blood oxygenation. A subset of rats in the low-dose DEX-KET group had early and transient reductions in SpO2 20 min post-treatment (Figure 4A), which can also occur in humans with rapid intravenous KET administration [64]. Nevertheless, these minor effects on blood oxygenation are likely preventable through preoxygenation or oxygenation during administration [65].

As expected due to the sympatholytic effects of DEX [66], heart rate was significantly reduced compared to the SAL control group in all treatment groups (Figure 4B). High-dose DEX, however, produced a significantly greater maximal reduction in heart rate compared to low-dose DEX ± KET, and this effect persisted throughout the entire 4-h trial. Low-dose DEX-KET hastened the return to baseline heart rate, taking 120 min compared to 180 min for low-dose DEX alone. In addition, the bradycardia in the combination group was less severe, as heart rate was significantly higher than in the low-dose DEX monotherapy group during the 20- and 60-min post-treatment measurements. This improved cardiovascular safety profile compared to low-dose DEX alone is attributable to the sympathomimetic effects of KET [40], which oppose DEX-induced reductions in cardiac output. The incomplete attenuation of DEX-induced cardiosuppression may provide a therapeutic benefit in attenuating METH-induced hypertension and tachycardia [7].

All three treatments resulted in both significant and substantial elevations in blood glucose levels. These levels largely recovered by the conclusion of the 4-h trial in the low-dose DEX ± KET groups, while they remained highly elevated after high-dose DEX treatment (i.e., only one rat out of eight had a blood glucose level lower than 200 mg/dL 4 h after high-dose DEX treatment, Figure 4C). While DEX-induced hyperglycaemia may not be observed in humans during surgery due to the attenuative effects of DEX on stress-induced hyperglycaemia [67], this effect has been observed in accidental exposure [68] and in non-surgical clinical use [69]. Considering these clinical observations outside of surgery, our experimental findings, and the general ease of monitoring this parameter, blood glucose should be monitored upon DEX ± KET administration in any future clinical trials of this novel intervention.

In the current study, there were significant reductions in temperature in all three treatment groups (Figure 4D). The hypothermic effect was maximal 60 min after treatment administration in the low-dose DEX ± KET groups and fully recovered at the conclusion of the 4 h trial. In the high-dose DEX group, temperatures continued to drop for up to 120 min post-administration to produce more intense hypothermia, which did not recover during the study period. In humans, DEX-induced hypothermia is also a common occurrence [70–73]. Considering the potential for DEX-induced thermal dysregulation, temperature should be monitored in any future clinical trials of this intervention.

As expected, the current study found that DEX dose-dependently and significantly increased urine production (Figure 5A) but unexpectedly resulted in a significant reduction in pH (Figure 5B). It should be noted that more acidic urine can result in the ion trapping of METH and related compounds in the renal tubule, thereby enhancing renal elimination [49, 50, 74]. While we observed a significant DEX-induced increase in the percentage of unchanged METH excreted in the urine in a small preliminary study, we only observed a trend (p = 0.06) towards this effect in the current study (Figure 5C). We suspect that this lesser effect was at least partially due to DEX polyuria-induced loss of urine during transport from the diuresis cages to testing sites at early post-treatment time points. This only occurred in 2/8 control rats but happened more frequently in 6/8 low-dose DEX-treated rats, 4/8 low-dose DEX-KET-treated rats, and 7/8 high-dose DEX-treated rats. To measure the effects of DEX-KET during the most intense METH-induced locomotor activity (Figure 2B), the first experimental time point in the preliminary study was moved from 5 to 20 min in the current study. Urine loss was less common in the preliminary study, as the first measurement was taken before the onset of intense DEX-induced polyuria. Nevertheless, the secondary analysis showed increasing volume (Figure 5D) and decreasing pH (Figure 5E) to have a moderate, significant correlation with the percentage of unchanged METH eliminated in the urine. Future evaluation of this potential effect will employ a dedicated pharmacokinetic study to avoid urine loss, alongside serum measurements to evaluate any potential beneficial effects on systemic METH exposure.

These data provide encouraging initial preclinical proof-of-concept for the safety and efficacy of using DEX-KET to produce deeper sedation than DEX alone in METH-induced agitation. Prior to the clinical development of this novel intervention, however, it is critical to conduct a more rigorous preclinical safety test in the context of opioid-stimulant co-exposure. Also, a preclinical comparison study with the benzodiazepine standard of care [7] is needed to demonstrate superiority for this indication. In addition, sex comparisons are important for further development, as female rats, similar to female humans, are more sensitive to the stimulant effects of METH [43, 75] and the sedative effects of DEX [76, 77] than males of each species. Unfortunately, the relevant sex/species effects of KET are less consistent, understood, and established. For example, female rats are more sensitive to KET-induced sedation than male rats [76], but an FDA Adverse Event Reporting System data pharmacovigilance study (limited by voluntary reporting bias, missing exposure denominator, and inclusion of data from both sedative and antidepressant indications) has suggested that sedation may occur more frequently in human males than females [78]. Should this effect in humans be verified in the future, it could be explained by higher systemic exposure to KET and its active metabolite norketamine in female rats compared to male rats [79] and in male compared to female humans [80, 81].

In this study, we showed that the combination of adjunctive KET and low-dose DEX can reduce the most intense period of METH-induced locomotor activity (i.e., the rat model of METH-induced agitation [22–24]), in a manner similar to that of high-dose DEX. In addition to the low-dose DEX-KET-induced sedation during this period being deeper than that induced by high-dose DEX, the adverse effects profile was also superior, with reduced bradycardia compared to low-dose DEX alone. Overall, these data on adverse effects based on clinically relevant endpoints may be useful in guiding the design of future clinical trials of this novel intervention. Fortunately, some DEX side effects may be beneficial in treating the common clinical features of severe METH intoxication, including tachycardia, hypertension, and hyperthermia, when not excessive [7]. The effectiveness of low-dose DEX in attenuating ongoing METH-induced locomotor activity also demonstrates the proof-of-concept of DEX administration after naloxone unmasking of opioid-induced sedation. This clinically relevant design was facilitated by a research team comprising individuals with expertise in clinical and preclinical research, pharmacy, veterinary medicine, and clinical toxicology, and is representative of our group’s strategy of designing clinically guided preclinical research to inform future clinical studies.

## Data Availability

The raw data supporting the conclusions of this article will be made available by the authors, without undue reservation.
