# Supplementary material for: Ketamine enhancement of dexmedetomidine attenuation of methamphetamine-induced agitation in rats
Source: J Pharm Pharm Sci. 2026 Apr 15;29:16294. doi: 10.3389/jpps.2026.16294 (PMC13124640; doi:10.3389/jpps.2026.16294)
Supplement: Supplementary file 1 [file DataSheet2.pdf]

<u>SUPPLEMENTAL FILE 2:</u> Distance traveled over time for individual rats

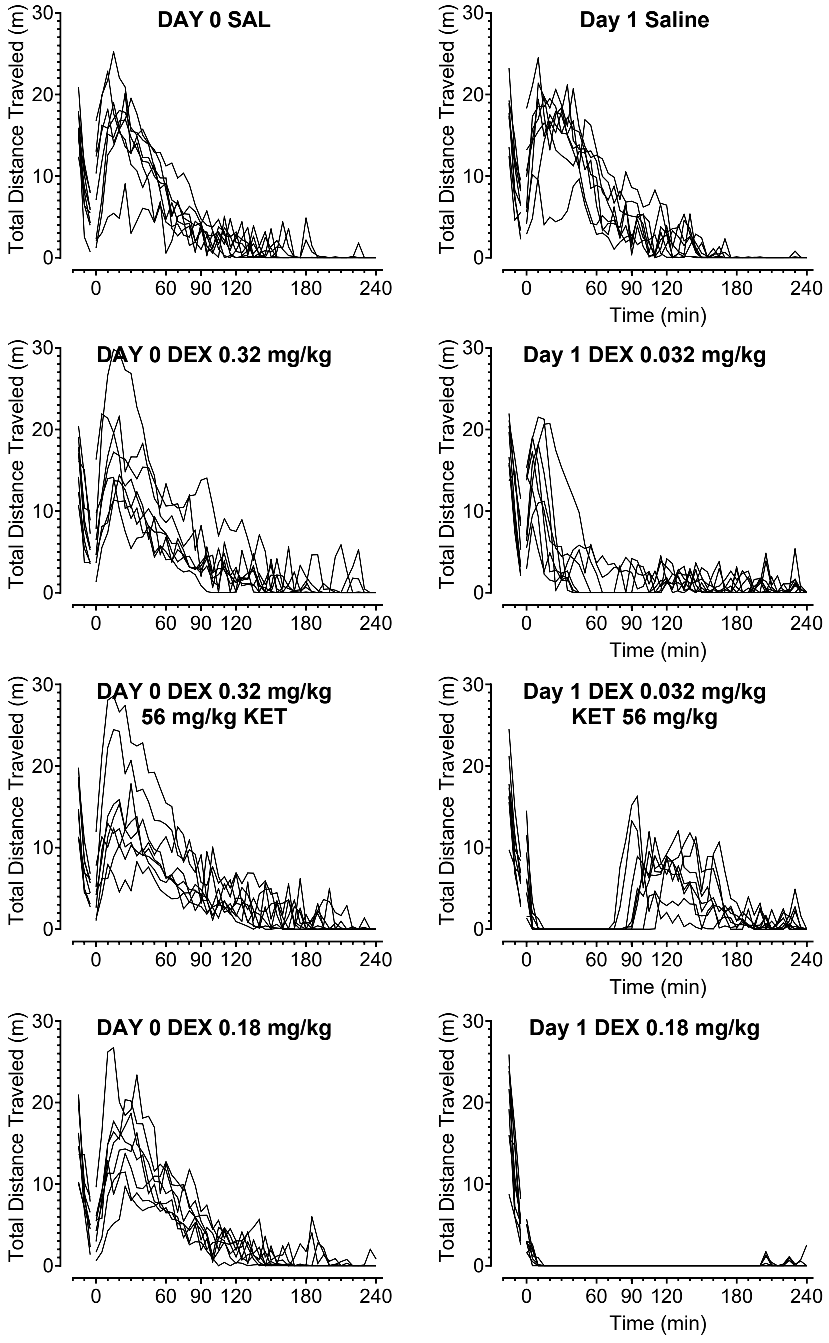

Supplemental Figure 2. Distance traveled over time for individual rats. Each line depicts 5 min activity intervals plotted for a single animal (symbols removed for clarity). The average plot for measured after each METH ± treatment exposure were combined on day 0 (Figure 1A) and day 1 (Figure 1B).
