# Supplementary material for: Ketamine enhancement of dexmedetomidine attenuation of methamphetamine-induced agitation in rats
Source: J Pharm Pharm Sci. 2026 Apr 15;29:16294. doi: 10.3389/jpps.2026.16294 (PMC13124640; doi:10.3389/jpps.2026.16294)
Supplement: Supplementary file 2 [file DataSheet1.pdf]

<u>SUPPLEMENTAL FILE 1:</u> Dexmedetomidine and ketamine dose verification

INTRODUCTION

In preliminary locomotor activity studies, male Sprague Dawley rats (n = 4 or 8/dose) were administered 1 mg/kg subcutaneous (SC) methamphetamine (METH) followed 15 min later by escalating doses of 0.0032, 0.01, 0.032, 0.56, 0.1, 0.14, and 0.18 mg/kg dexmedetomidine (DEX) on non-subsequent days. The 0.032 mg/kg dose was needed to produce a significant reduction in METH-induced locomotor activity, and the 0.18 mg/kg dose was needed to completely attenuate activity in the 90-min post-treatment time period of interest (i.e., during the most intense METH-induced activity). Note that 0.14 mg/kg DEX produced intense, but incomplete early sedation. Afterward, the minimally effective DEX dose was tested in combination with escalating doses of 10, 32, 56, and 100 mg/kg ketamine (KET). The addition of 56 mg/kg KET completely attenuated activity during the majority of the 90-min post-treatment period of interest, while producing less post-sedation emergence activity than the 100 mg/kg dose. Treatment of METH-intoxicated rats with 56 mg/kg KET in the absence of DEX resulted in intense stereotypy followed by an enhanced peak locomotor response. A qualitative dose verification study was performed to verify the subtherapeutic (0.01 mg/kg), minimally effective (0.032 mg/kg), and completely effective (0.18 mg/kg) DEX doses and the most completely sedating DEX adjunctive KET dose (56 mg/kg) determined during the preliminary study as well as our rationale for the exclusion of 56 mg/kg KET monotherapy from the preclinical trial.

MATERIALS AND METHODS

Drugs

A 1 mg/kg METH ([S]-methamphetamine HCl; Sigma Aldrich, St. Louis MO) solution was prepared in saline (SAL) for administration at 1 ml/kg and stored under refrigeration to maintain stability. DEX hydrochloride (veterinary grade, 0.5 mg/ml; Dechra, Cheshire CT) was diluted in saline (SAL) to 0.001, 0.032, or 0.18 mg/ml for SC administration at 1 ml/kg. KET HCl (veterinary grade, 100 mg/ml; Covetrus, Portland ME) was diluted with SAL to 56 mg/ml for SC administration at 1 ml/kg. The DEX and KET dilutions were prepared just prior to each experiment as the stability of the diluted agents is unknown.

Animals

Male Sprague Dawley rats (Hilltop Laboratory Animals, Scottsdale PA) were approximately 8 weeks old on study day 0 (n = 8, 4/treatment group). Rats were dual housed in a room held at 21 to 22°C and 40-55% humidity with *ad libitum* food and water provided. The animal use protocol was approved by the Marshall University Institutional Animal Care and Use Committee (Protocol #855) and was performed in compliance with the National Institutes of Health (NIH) Guide for the Care and Use of Laboratory Animals and the ARRIVE guidelines.

Experimental Design

Rats were conditioned to the researchers and the towel used for gentle restraint during SC injection on Days -4 and -3. On day 0, rats were SC administered 1 mg/kg METH prior to 14 min of locomotor activity measurements in a 74 cm tall x 58 cm wide x 58 cm long open field chamber with the Noldus EthoVision 14 automated behavioral analysis system (Noldus Information Technology Inc, Sterling VA). The animals were then removed from the chamber and SC injected with saline at two separate sites prior to an additional 3.5 hr of locomotor activity measurements. Using the total distance traveled data from the day 0 trial, rats were divided into groups of similar average activity (n = 4/group). The day 0 experiment was repeated in both groups on days 1, 2, 3, 8, 9, and 10 with the control group administered saline and the treatment group administered escalating doses of DEX (0.01, 0.032, and 0.18 mg/kg) plus SAL at a second site on days 1, 2, and 3; the minimally effective DEX dose plus 32 and 56 mg/kg KET on days 8 and 9; and SAL plus 56 mg/kg KET alone on day 10. The distance traveled in 5 min intervals over time data output was used to qualitatively verify doses chosen.

RESULTS AND DISCUSSION

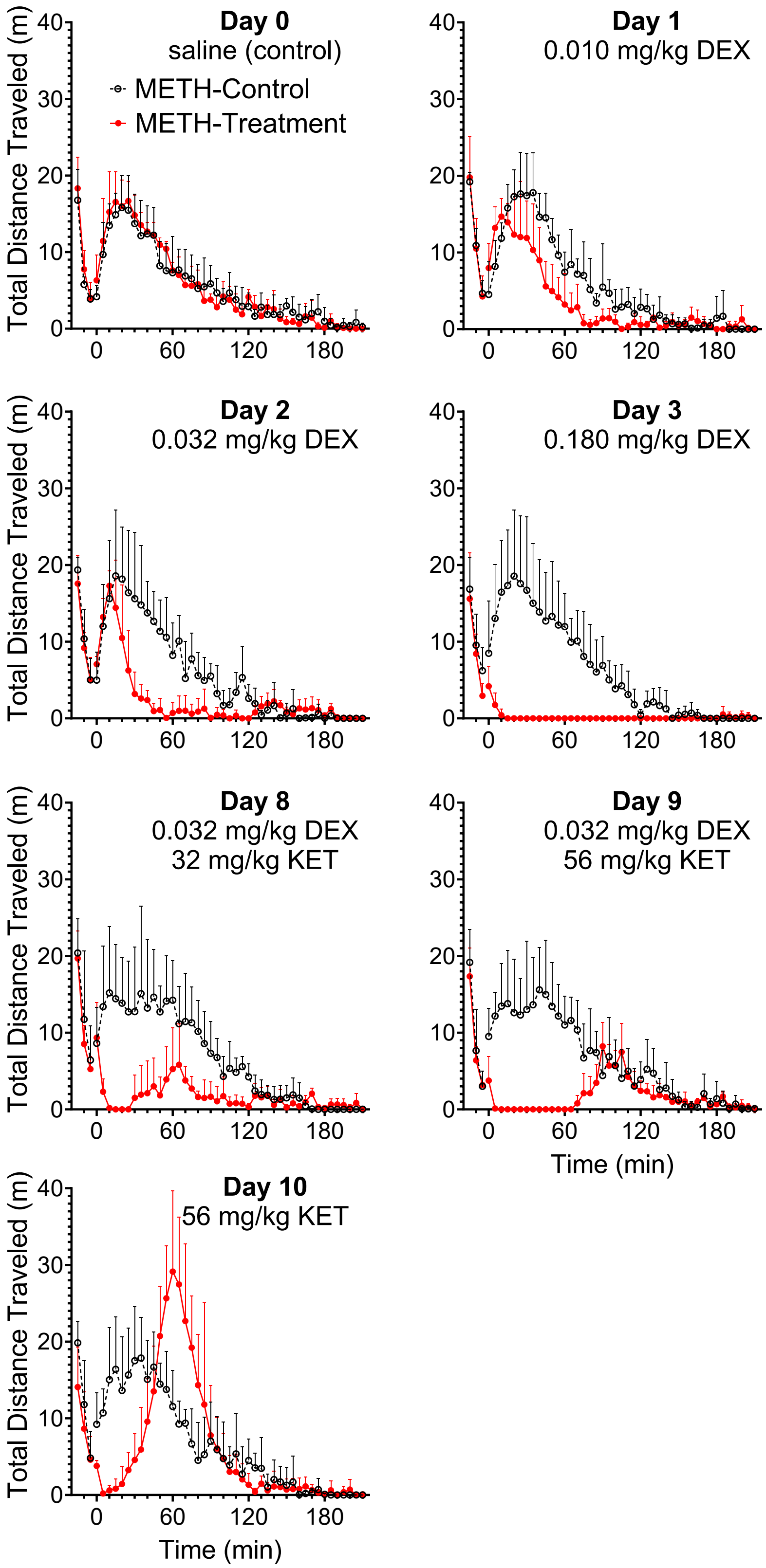

Supplemental Figure 1. Distance traveled over time (n = 4/group). Average activity over time + SD is plotted in 5 min intervals from METH injection at -15 min to SAL control or treatment injection at time 0 and for an additional 210 min. Each plot is labeled with the experiment day and treatment administered.

The overlapping locomotor activity over time data between groups on day 0 demonstrates the effectiveness of matching into groups with similar baseline METH-induced locomotor activity while the subtherapeutic 0.01 mg/kg DEX dose on day 1 produced a slight decrease in activity. Similar to the preliminary experiments, METH-induced locomotor activity was suppressed substantially after the minimally effective 0.032 mg/kg DEX dose on day 2 and almost completely after the high 0.18 mg/kg DEX dose on day 3. While the addition of 32 mg/kg KET to 0.032 mg/kg DEX on day 8 accelerated the onset of sedation and increased its overall intensity, the day 9 dose of 56 mg/kg KET was needed to completely attenuate METH-induced activity during the majority of the 90-min post-treatment period. The day 10 test of 56 mg/kg KET monotherapy initially produced intense stereotypy which was followed by an intense locomotor effect with peak activity substantially greater than that produced by METH alone.

The locomotor data collected verified the DEX and KET doses selected for testing in the preclinical trial of DEX-KET for deeper sedation in METH-induced agitation. Since the day 10 test of KET alone appeared to enhance the effects of METH, it was excluded from the preclinical trial.
